# 1-Pbps orbital angular momentum fibre-optic transmission

**DOI:** 10.1038/s41377-022-00889-3

**Published:** 2022-07-05

**Authors:** Junyi Liu, Jingxing Zhang, Jie Liu, Zhenrui Lin, Zhenhua Li, Zhongzheng Lin, Junwei Zhang, Cong Huang, Shuqi Mo, Lei Shen, Shuqing Lin, Yujie Chen, Ran Gao, Lei Zhang, Xiaobo Lan, Xinlun Cai, Zhaohui Li, Siyuan Yu

**Affiliations:** 1 State Key Laboratory of Optoelectronic Materials and Technologies, School of Electronics and Information Technology, Sun Yat-Sen University, Guangzhou, 510006 China; 2 Yangtze Optical Fibre and Cable Joint Stock Limited Company, State Key Laboratory of Optical Fibre and Cable Manufacture Technology, No.9 Guanggu Avenue, Wuhan, Hubei China; 3 School of Information and Electronics, Beijing Institute of Technology, Beijing, 100081 China

**Keywords:** Fibre optics and optical communications, Applied optics

## Abstract

Space-division multiplexing (SDM), as a main candidate for future ultra-high capacity fibre-optic communications, needs to address limitations to its scalability imposed by computation-intensive multi-input multi-output (MIMO) digital signal processing (DSP) required to eliminate the crosstalk caused by optical coupling between multiplexed spatial channels. By exploiting the unique propagation characteristics of orbital angular momentum (OAM) modes in ring core fibres (RCFs), a system that combines SDM and C + L band dense wavelength-division multiplexing (DWDM) in a 34 km 7-core RCF is demonstrated to transport a total of 24960 channels with a raw (net) capacity of 1.223 (1.02) Peta-bit s^−1^ (Pbps) and a spectral efficiency of 156.8 (130.7) bit s^−1^ Hz^−1^. Remarkably for such a high channel count, the system only uses fixed-size 4 × 4 MIMO DSP modules with no more than 25 time-domain taps. Such ultra-low MIMO complexity is enabled by the simultaneous weak coupling among fibre cores and amongst non-degenerate OAM mode groups within each core that have a fixed number of 4 modes. These results take the capacity of OAM-based fibre-optic communications links over the 1 Pbps milestone for the first time. They also simultaneously represent the lowest MIMO complexity and the 2nd smallest fibre cladding diameter amongst reported few-mode multicore-fibre (FM-MCF) SDM systems of >1 Pbps capacity. We believe these results represent a major step forward in SDM transmission, as they manifest the significant potentials for further up-scaling the capacity per optical fibre whilst keeping MIMO processing to an ultra-low complexity level and in a modularly expandable fashion.

## Abstract (web summary)

A 1-Pbps orbital-angular-momentum fibre-optic transmission system that combines space-division multiplexing and wavelength-division multiplexing in a 34 km 7-core ring-core fibre is demonstrated utilizing low-complexity 4 × 4 MIMO equalization.

## Graphical abstract

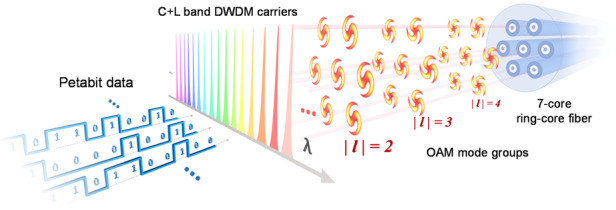

## Introduction

Over the past 40 years, a series of technical breakthroughs such as erbium-doped fibre amplifier (EDFA), wavelength-division multiplexing (WDM), coherent optical detection and high-order modulation formats, etc. have pushed the capacity per single mode fibre (SMF) rapidly towards its nonlinear Shannon limit^1^. Meanwhile, space-division multiplexing (SDM) techniques, that explore the degrees of freedom in the transverse spatial domain by means of few-mode fibres (FMFs), multi-core fibres (MCFs), and few-mode multi-core fibres (FM-MCFs), have been widely investigated with dramatic progress being made in achieving multi-petabit per second throughputs over several tens or even more than one hundred spatial channels^2–11^ per fibre.

In fibres supporting ultra-high data throughput over large numbers of closely packed or even overlapping spatial channels, the crosstalk among spatial channels, arising due to their optical coupling, is one of the major problems that would have to be dealt with by large-scale multi-input multi-output (MIMO) digital signal processing (DSP). In addition to the number of coupling spatial channels, the MIMO complexity in SDM systems is also exacerbated by the differential group delay (DGD) among the coupled optical channels, as each optical data symbol in one channel is coupled to all other data symbols in all other channels within the temporal vicinity of the DGD. If not managed properly, the rapidly increasing computational complexity associated with such DSPs can be detrimental to the scalability of SDM towards higher capacity.

**Fig. 1:**
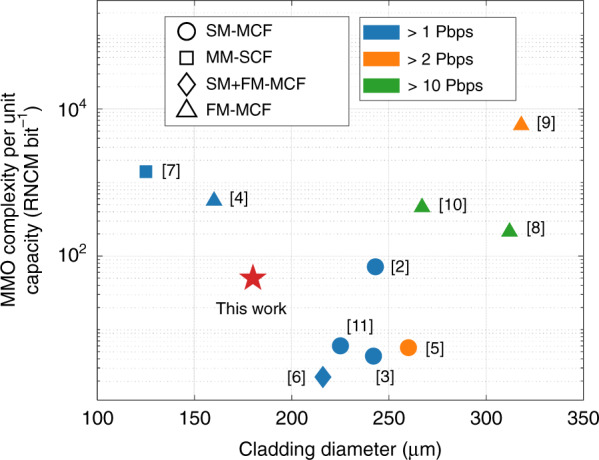
MIMO complexity vs. cladding diametre. The data points are evaluated based on the information reported from the cited references that demonstrated over 1 Pbps capacity SDM transmission.

Figure 1 summarizes the MIMO complexity per unit capacity, expressed as the required number of complex multiplications (RNCM) per bit^12,13^ (calculation details can be found in Supplement S1) versus the fibre cladding diametres in recent SDM/WDM experimental transmission systems with single-fibre capacity over 1 Peta-bit s^−1^ (Pbps). It becomes apparent that low MIMO complexity is generally only achieved at large cladding diametres. This general trade-off is underlined by the increasing optical coupling between the spatial channels (i.e., different cores and different modes within each core) at higher channel numbers and packing densities. Systems utilizing single-mode (SM) MCFs^2,3,5,11^ or FM-MCFs^4,8–10^ can effectively alleviate MIMO complexity as long as weak inter-core coupling is maintained by keeping the cores sufficiently apart so that MIMO equalization is only required to compensate the inter-mode crosstalk in each fibre core, thereby reducing the size of the MIMO matrix to be processed. However, a high number of multiplexed spatial channels, as well as low inter-core coupling require enlarged fibre cladding diametres of more than 200 μm^2,3,5,6,8–11^, which will degrade performance in fibre fabrication, splicing, and reliability^14^. Even with negligible inter-core coupling, current high-capacity FM-MCF SDM systems, in order to deal with intra-core modal crosstalk arising from both the in-fibre mode coupling and the non-ideal spatial/mode (de)multiplexers, still require MIMO equalization that covers the full mode set supported by each core. For example, 6 × 6 or even 12 × 12 MIMO equalizations with hundreds of time-domain equalization (TDE) taps are demanded when 3 or 6 modes are supported in each fibre core of the FM-MCFs^4,8–10^. Simultaneous low channel coupling among fibre cores and amongst the modes/mode groups (MGs) within each core are therefore called for in FM-MCF transmission links where the number of multiplexed channels can be expanded by increasing mode channels per core, rather than the number of fibre cores, to keep fibre cladding diametre small and allow utilization of small-scale modular MIMO processing with a complexity not only very low but also not growing with the number of modes. It has been challenging, however, for fibre cores with conventional step- or graded-index refractive index profiles (RIPs) to achieve such an objective.

Since its first demonstration^15^, ring-core-fibre (RCF)-based optical communication systems that utilize orbital angular momentum (OAM) modes have received sustained attention as a potential candidate for improving the capacity of SDM systems with low DSP complexity^16–22^. In a RCF, with the radial confinement of the ring-shape core, the number of near-degenerate modes in OAM MGs characterized by their common topological charge value*l*can be made constant at 4 per MG when |*l*| ≥ 1 (four modes in each OAM MG |*l*| ≥ 1: OAM modes with topological charge value +*l* and *−l* each carrying two orthogonal polarizations)^23,24^. The differential effective refractive index ∆*n*_eff_ between adjacent MGs increases with |*l*|^17,20,25^, which leads to reduced inter-MG coupling therefore good scalability to high-order mode space. It was also shown that, by engineering the RIP of the RCF^26,27^, it is possible to further split the spin-orbital aligned and anti-aligned sub-groups within each MG of the same |*l*|, so that the two sub-groups propagate with relatively low coupling and act as independent channels up to certain distances^28–30^. In addition to the beneficial mode-coupling characteristics during fibre transmission, efficient OAM mode sorting can also be achieved by only having to consider the azimuthal dimension in a relatively simple mode transform process^31,32^, which is conducive to realizing spatial/mode (de)multiplexing modules that simultaneously achieve low crosstalk among fibre cores and amongst the modes/MGs within each core.

With these merits, RCF-based OAM mode multiplexed transmissions over single-span fibre of 1 km^15,29,33^, 10 km^20^, 18km^21,22^, 24 km^34^, 50 km^18^, and 100 km^19^ have been achieved with aggregated capacity up to 10-Tbps level utilizing only modular 4 × 4 MIMO or MIMO-free processing (detailed parametres of the transmission systems are summarized in Table S3 of Supplement S2). However, to the best of our knowledge, OAM mode based SDM transmissions with capacity over 1 Pbps have not yet been reported.

**Fig. 2:**
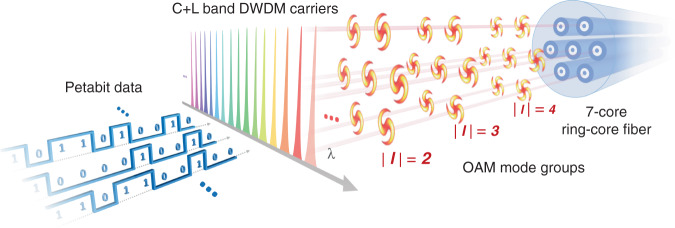
An illustration of the 1Pbps OAM-SDM-WDM multiplexing scheme. The system combines the C+L band WDM channels, spatial/mode multiplexing as well as the 34-km 7-core RCF to realize 1Pbps transmission.

This paper reports the experimental demonstration of a 1.02 Pbps OAM mode based SDM transmission system that only requires 4 × 4 MIMO processing with TDE tap number no more than 25, which, to the best of our knowledge, is the lowest MIMO complexity in FM-MCF based SDM transmission demonstrations above 1 Pbps. As illustrated in Fig. 2, within a cladding diametre of 180 µm, the 34 km 7-core RCF affords a total of 80 available OAM mode channels (6 cores × 6 OAM modes × 2 orthogonal polarizations + 1 core × 4 OAM modes × 2 orthogonal polarizations). Weak coupling among fibre cores and amongst the non-degenerate OAM MGs within each core has been simultaneously realized for the entire transmission link consisting of both the optical fibre and spatial/mode (de)multiplexing modules, leaving only the coupling among the 4 near-degenerate modes within each MG to be dealt with by the very small-scale 4 × 4 modular MIMO. Capacity expansion is realized by simply using more OAM MGs and the same 4 × 4 MIMO modules. In each OAM mode channel, 312 WDM channels are transmitted covering the C and L band with 25 GHz spacing and each carrying 24.5-GBaud quadrature phase shift keying (QPSK) signals, achieving a raw (net) capacity of 1.223 (1.02) Pbps and a spectral efficiency (SE) of 156.8 (130.7) bit s^−1^ Hz^−1^ with the bit-error rate (BER) below the 20% soft decision forward error correction (FEC) threshold of 2.4 × 10^−2^.

These results take the capacity of OAM-based fibre-optic communications links over the 1 Pbps milestone for the first time. They also simultaneously represent the lowest MIMO complexity and the 2nd smallest fibre cladding diameter amongst reported FM-MCF-based SDM systems of >1 Pbps capacity. Therefore, the results manifest the significant potentials for further up-scaling the capacity per optical fibre by exploiting the OAM modes in optical fibres to keep MIMO processing to an ultra-low complexity level and in a modularly expandable fashion.

### Experimental setup of the OAM-SDM-WDM transmission

**Fig. 3:**
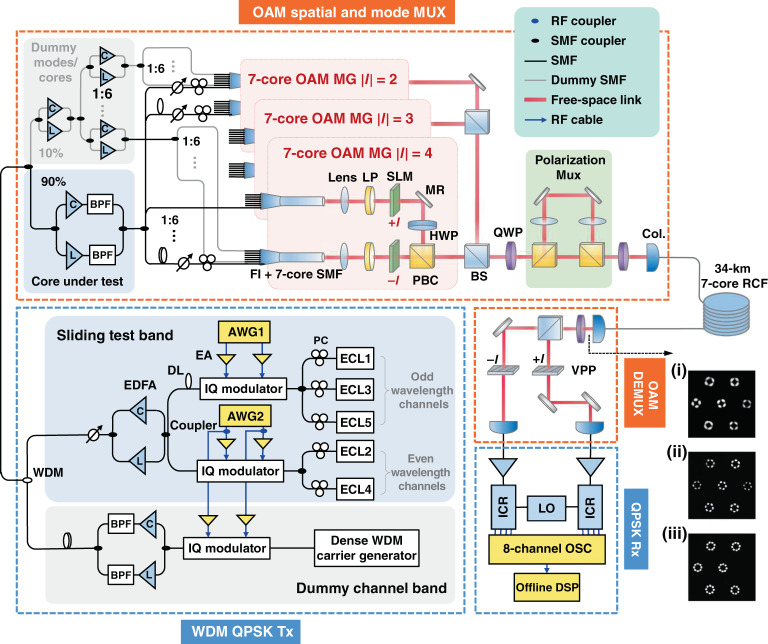
The experimental setup for OAM-SDM-WDM data transmission. The intensity profiles of OAM MGs |*l*| = (i) 2, (ii) 3, and (iii) 4. A linear polarization (LP)-mode-like intensity profile in each fibre core is observed due to the coherent superposition of the four intra-MG OAM modes^17^. ECL external cavity laser; BPF band-pass filter; OP optical processor; FI Fan-in device; EDFA erbium-doped fibre amplifier; LP linear polarizer; SLM spatial light modulator; MR mirror; HWP half-wave plate; Col. collimator; VPP vortex phase plate; ICR integrated coherent receiver; OSC oscilloscope; LO local oscillator; RF radio frequency; DSP digital signal processing

Figure 3 illustrates the setup for the 1 Pbps OAM-SDM-WDM data transmission experiment, which mainly consists of five parts: a WDM signal generator with QPSK modulation, a 7-core OAM spatial and mode multiplexing (MUX) module, the 34 km 7-core RCF, an OAM mode demultiplexing (DEMUX) module and coherent optical receivers followed by DSP including the offline 4 × 4 MIMO equalization.

### The transmitter including WDM signal generation and OAM-SDM MUX

At the transmitter, a high optical signal-to-noise ratio (OSNR) sliding test channel band and a low OSNR dummy channel band are implemented simultaneously to produce the 312 WDM channels using limited device resources. Five optical carriers from wavelength-tuneable external cavity lasers (ECLs) with wavelengths at a 0.2 nm/25 GHz grid are used as the test band, which are divided into even and odd groups and independently modulated by two 24.5-GBaud QPSK signals (the detailed parametres of the electrical QPSK signals can be found in the section of “Materials and methods”) from a four-channel arbitrary waveform generator (AWG) through in-phase/quadrature (I/Q) modulators. The generated optical signals of the odd and even groups are further de-correlated using an optical delay line (DL) to avoid performance overestimation of the WDM system^35^. The resulting 5-channel test band is amplified by a C or L band EDFA, as the central wavelength of the test band is adjusted to align with the WDM channel under test. A variable optical attenuator is utilized after the EDFAs to control the optical power of the test band. A dense WDM carrier generator realized through multiple seed light sources modulated by cascaded Mach–Zehnder modulators and phase modulators^36^ is utilized as the light source of the dummy band. After being modulated by the 24.5-Gbaud QPSK signals, the dummy band is combined with the test band and subsequently amplified employing C and L band EDFAs to form WDM signals with 312 channels ranging from 1538.19 to 1602.10 nm. There is no gap between the C and L bands of the WDM signals because the actual response bandwidths of the C and L band EDFAs used in our experiment can be partially overlapped and cover the whole C and L optical-wavelength band (see optical spectra shown in Fig. 6d).

The generated WDM signals are subsequently split into two branches via a 1:9 optical power splitter. 90% optical power is used to generate mode channels with high OSNR in the fibre core under test, while the remaining 10% optical power, which is further amplified by high-power C and L band EDFAs, is used to implement low OSNR channels in the dummy cores. After passing through the EDFAs, power splitters and optical DLs, the produced test and dummy spatial/mode channels are directed to the associated input port of the 7-core OAM mode multiplexing module (whose details can be found in the section of “Material and methods”), to generate hexagonally packed OAM multiplexed beams with topological charge *l* = ±2, ±3, and ±4. After propagating through the polarization multiplexing module with a 4-f free-space de-correlation path (details are presented in the section of ‘material and methods’), the 80 spatial/mode channels [(6 cores × 6 OAM modes + 1 core × 4 OAM modes) × 2 orthogonal circular polarizations] each carrying 312 wavelengths are generated and finally coupled into the 7-core RCF. Here 80 rather than 84 spatial/mode channels are used because the OAM MG |*l*| = 4 in Core #2 cannot be supported after 34 km transmission (see inset iii in Fig. 3). OAM modes with topological charge *l* = 0 (i.e., the fundamental modes) and *l* = ±1 (in the OAM MG |*l*| = 1) are not utilized in the OAM SDM transmission since they are strongly coupled after 34 km fibre transmission due to their small effective refractive index difference (Δ*n*_eff_) (details can be found in Supplement S3). Thus 6 × 6 MIMO equalization should be required to compensate crosstalk among them (3 OAM modes × 2 polarizations), which could not be realized experimentally due to the equipment resource limitation in our laboratory.

### The Receiver including OAM DEMUX and coherent detection

After 34-km fibre transmission, the 7-core OAM multiplexed beams are received for mode demultiplexing, as the intensity profile of the OAM MGs |*l*| = 2, 3, and 4 are shown in the insets of Fig. 3. An LP-mode-like intensity profile in each fibre core is observed due to the coherent superposition of the four intra-MG OAM modes^17^. The distinctive and clear azimuthal distributions of the received intensity profiles indicate low inter-MG coupling in each fibre core. Then the beams are collimated, converted back to linearly polarization using a quarter-wave plate (QWP), and split into two branches. The OAM beam of the target spatial/mode channel under test in each of the two branches is converted into a Gaussian beam through a vortex phase plate (VPP) and subsequently coupled into an SMF-pigtailed dual-polarization integrated coherent receiver (ICR). Due to equipment resource limitations on the receiver side, only one group of 4 OAM mode channels in the fibre core under test are simultaneously de-multiplexed and received to go through the 4 × 4 MIMO equalization that compensates the intra-MG modal crosstalk. Signals in different OAM MGs/fibre cores are asynchronously de-multiplexed, received, and tested one by one. The electrical waveforms from the ICRs are simultaneously sampled and stored by an eight-channel real-time oscilloscope operated at a sampling rate of 50 GSa s^−1^ for offline DSP and BER evaluation. The DSP algorithms mainly include timing phase recovery, 4 × 4 MIMO equalization based on the conventional blind constant modulus algorithm (CMA), frequency offset compensation, and carrier phase estimation. This measurement is repeated for each OAM MG, fibre core as well as wavelength channel and finally, BERs of all the 24960 channels are evaluated.

## Results

### Characteristics of the 34 km 7-core RCF

**Fig. 4:**
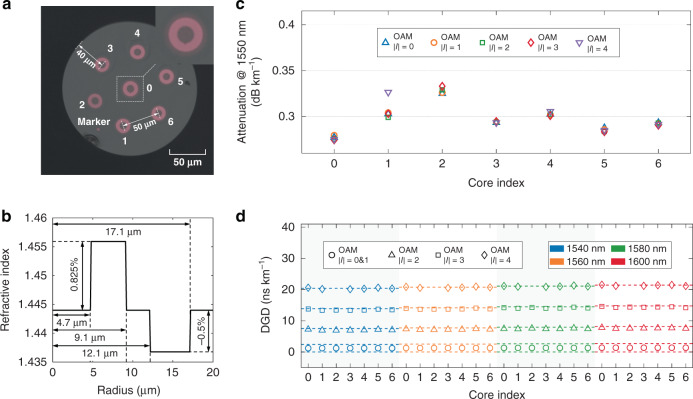
The characteristics of the 34 km 7-core RCF. **a** The cross-section diagram; **b** The refractive index profiles; **c** The measured mode dependent attenuation of 7-core RCF at 1550 nm; **d** The measured and simulated DGD at 1540, 1560, 1580, and 1600 nm of the designed fibre (dashed lines represent the simulated results). One averaged DGD value is given for OAM MGs |*l*| = 0 & 1 in the measured results, as their impulse response merged into one Gaussian-distribution peak due to strong coupling between these two MGs after 34 km transmission

The cross-section of the homogeneous 7-core RCF is shown in Fig. 4a, which was specially designed and fabricated to enable simultaneous weak coupling among fibre cores and among the non-degenerate OAM MGs within each core. The parametres of the overall cross-sectional geometry and the RIP of each core are provided in Fig. 4a, b, respectively. The calculated effective refractive index *n*_eff_ of all the guided modes across the C and L band can be found in Supplement S3.

The attenuation of all guided MGs at the wavelength of 1550 nm is measured by means of optical time-domain reflectometry as detailed in Supplement S4. As shown in Fig. 4c, there is nearly no MG-dependent attenuation in the cores of #0, #3–#6, while in the fibre core #1 a small difference of 0.02 dB km^−1^ exists due to the slightly higher attenuation of the highest order OAM MG with topological charge *|l*| = 4. As for the fibre core #2, the OAM MG |*l*| = 4 cannot be supported as a low-loss guided MG mainly due to fibre structure deformation during the drawing process. The MG- or core-dependent attenuation of the 7-core RCF, however, has little impact on the MIMO equalization performance, as only modular 4 × 4 MIMO equalization is required to compensate the intra-MG mode crosstalk and the 4 near-degenerate intra-MG modes are strongly coupled with no tangible differential attenuation^19^. The fibre attenuation variation across the WDM transmission band is also evaluated utilizing a cut-off method, as detailed in Supplement S5. The wavelength-dependent attenuation variation is no more than 0.02 dB km^−1^ over the wavelength range from 1538 to 1602 nm.

The DGD of the guided MGs at four wavelengths ranging from 1540 to 1600 nm with 20 nm spacing are measured with a vector network analyzer (VNA)^37^ using a time-domain impulse response method, detailed in Supplement S6. As shown in Fig. 4d, large DGD with values of more than 5 ns km^−1^ between adjacent pairs of OAM MGs of topological charge *|l*| ≥ 1 can be achieved in each fibre core for all four selected wavelengths. The large inter-MG DGD, totalling ~ 20 ns km^−1^ or 680 ns over the length of the link, serves to maintain low optical coupling among these higher-order MGs but does not impact the tap count of the DSP.

### Power budget evaluation of the OAM-SDM-WDM system

The power budget of the OAM-SDM-WDM experiment system is evaluated, as the results shown in Table 1. The average power of WDM signals at the input port of the fan-in device is set to 25–26 dBm (approximately 0.1 to 1.1 dBm per WDM channel), changing with OAM MG orders, due to their different insertion loss through the 7-core OAM MUX module. The total insertion loss of the OAM MUX module consists of both their optical element losses (e.g., the loss of the 3 dB beam combiner) and the coupling loss to the 7-core RCF. The measured coupling loss to 7-core RCF of OAM MG |*l*| = 4 is around 7 dB, 3 dB (2 dB) higher than that of the OAM MG |*l*| = 2 (|*l*| = 3). Such MG-order dependent loss may have resulted from the radial mismatch between the generated free-space OAM beams and the OAM modes supported in the 7-core RCF^38^, as well as the alignment errors of the optical elements used for fibre mode coupling, which can be improved by proper local phase modulation for OAM modes with different topological charge |*l*| in each fibre core and more precise optical-element aligning. One more 3 dB optical beam combiner is used in the optical propagating paths of the OAM beams with topological charge |*l*| = 2 and 3, respectively, compared with that for OAM beams with topological charge |*l*| = 4, as shown in Fig. 3, to balance the total insertion loss of the OAM MUX module among different OAM MGs. The total optical power of all the mode & wavelength channels launched to one fibre core is less than 20 dBm and there is no obvious nonlinear effect to significantly aggravate the BER performance in this case, according to the experimental evaluations shown in Supplement S7. After passing through the 34 km 7-core RCF and the OAM DEMUX module, ~ −32 dBm of optical power is received at the input port of the optical pre-amplifier located before the ICR, higher than the sensitivity (~ −37 dBm) of the pre-amplifier (details of the measured BERs versus the receiving sensitivity can be found in Supplement S8).

**Table 1:** Optical power budget evaluation of the OAM-SDM-WDM experiment systems.

OAM mode group	|*l*| = 2	|*l*| = 3	|*l*| = 4
Average power at Fan-In input	25 dBm	26 dBm	25 dBm
Average power per wavelength at Fan-In input	0.06 dBm	1.06 dBm	0.06 dBm
Insertion Loss of the OAM Mux. Module (including coupling loss)	13 dB	14 dB	13 dB
Average fibre loss/core	10.09 dB	10.10 dB	10.07 dB
Insertion loss of OAM Demux. Module (including coupling loss)	9 dB	9 dB	9 dB
Average received power before Pre-amp. EDFA for each fibre core	−32.03 dBm	−32.04 dBm	−32.01 dBm

### Transmission system crosstalk characterization

**Fig. 5:**
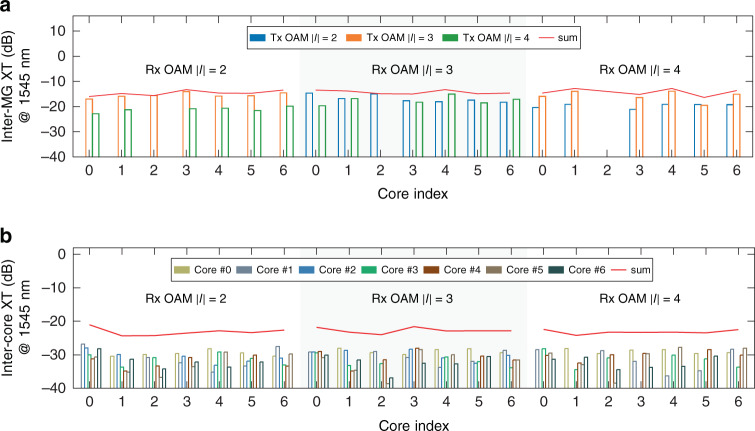
The measured crosstalk of OAM MGs |*l*| = 2, 3, and 4 after 34 km 7-core RCF transmission. **a** The measured inter-MG crosstalk within the same fibre core at 1545 nm; **b** The inter-core crosstalk at 1545 nm. For each core, the XT is contributed by all other 6 cores

The inter-MG crosstalk (XT) within the same fibre core, as well as the inter-core XT of the transmission system that consists of the 34 km 7-core RCF, the OAM MUX and DEMUX modules (orange dashed box in Fig. 3), is experimentally characterized, whose measurement details can be found in Supplement S9. As shown in Fig. 5a, the aggregated crosstalk among OAM MGs *|l*| = 2, *|l*| = 3 and *|l*| = 4 in the same fibre core is below −12 dB at wavelength of 1545 nm. The inter-MG crosstalk of each fibre core across the WDM transmission band is also evaluated by implementing measurement at selected wavelengths over C and L bands (1555–1585 nm with 10 nm spacing). The detailed results are illustrated in Supplement S9, which shows that all the measured aggregated inter-MG XTs at these wavelengths are below −12 dB, quite similar to the values measured at 1545 nm. Of this system XT, the contribution by the 34 km RCF only (excluding the XT from the MUX and DEMUX modules) is ~ −16 dB (around −31 dB km^−1^, details of the experimentally measured results are shown in the Supplement S9), indicating that the main source of the system XT is the OAM MUX and DEMUX modules. The relatively high XT of the OAM MUX results from a compromise when aligning the optics to achieve more equalized coupling loss to the 7-core RCF of each fibre core and each MG.

As shown in Fig. 5b, the aggregated inter-core XT from all other six cores at the wavelength of 1545 nm is less than −20 dB (red lines in Fig. 5b), around 8 dB lower than the inter-MG XT in the same fibre core. Such low levels of aggregated inter-core XT can also be maintained at wavelengths ranging from 1555 to 1585 nm, as the results shown in Supplement S9. Therefore, compared with the inter-core XT, the inter-MG XT in the same core dominates the system performance.

### BER evaluation

**Fig. 6:**
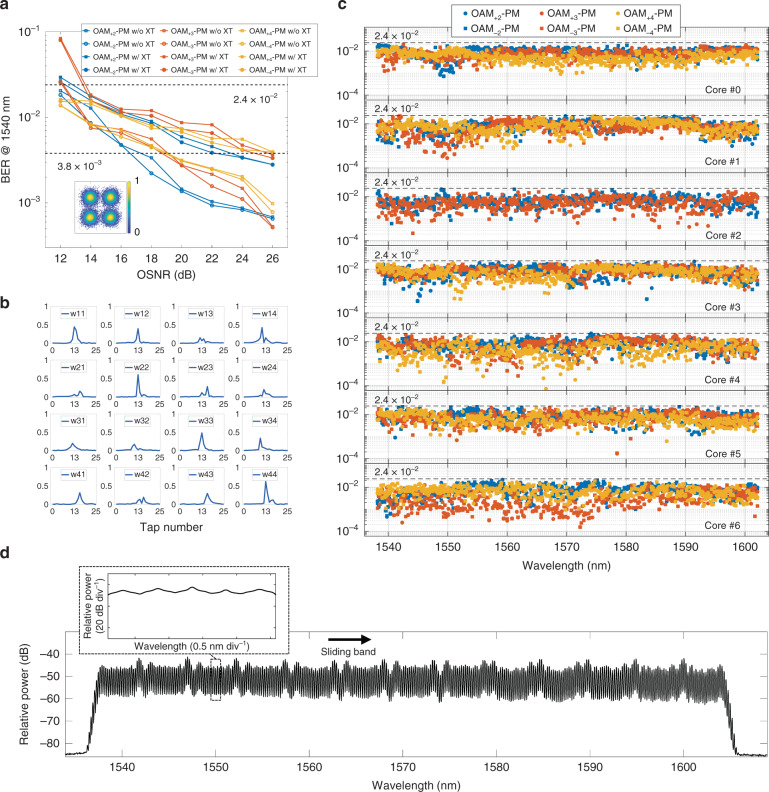
The characterization of the communication system. **a** The measured BER vs. OSNR curves at 1540 nm; **b** The absolute values of tap weights in 16 FIR filters of 4 × 4 MIMO equalizers to equalize the four modes belonging to OAM MGs *|l*| = 3 at 1540 nm; **c** The measured BERs of all 24960 channels after 34 km OAM-SDM-WDM transmission; **d** An optical spectrum of WDM signals with 312 wavelengths, inset: sliding test channel band

Before WDM transmission, a single-wavelength transmission scenario is firstly conducted to evaluate the BER values at different OSNR. The measured curves for OAM mode channels in the central core (Core #0 in Fig. 4) at the wavelength of 1540 nm are shown in Fig. 6a. Here the BER values of the two orthogonal polarization channels with the same OAM topological charge *l* are averaged for simplicity. It can be seen that when OSNRs are over 14 dB, BER below the 20% soft-decision FEC threshold of 2.4 × 10^−2^ can be achieved for all the OAM channels with the presence of the inter-core and inter-MG XT. There is an average 7 dB OSNR penalty between the cases with and without inter-core/inter-MG XTs for all the three OAM MGs *|l*| = 2–4 at the BER of 3.8 × 10^−3^ (7% hard-decision FEC threshold). Such OSNR-penalty values become around 8 to 9 dB for other selectively measured fibre cores (Core #6) / wavelength (1600 nm) channels, whose results are presented in Supplement S10. It should be emphasized that this BER performance is achieved using only 4 × 4 MIMO equalization modules. As a typical case, tap-weight absolute values of the 16 finite impulse response (FIR) filters to compensate intra-MG modal crosstalk of OAM MGs *|l*| = 3 in Core #0 at 1540 nm are illustrated in Fig. 6b. It can be seen that only 25 taps in each time-domain FIR filter are required due to the high degeneracy and strong coupling among the intra-MG modes. Tap-weight absolute values of the 4 × 4 MIMO equalization modules to recover intra-MG mode channels of other ring cores and wavelengths can be found in Supplement S11.

Figure 6c illustrates the measured BER values of all 24960 channels in the WDM transmission scenario, while the optical spectrum of the 312 WDM channels over the C and L band on the transmitter side is shown in Fig. 6d. The unevenness of the WDM spectrum is mainly due to non-ideal electro-optic modulation and the overlap between edges of adjacent sub-bands in the optical comb generation, which can be further flattened by means of a programmable optical processor (OP). Such uneven optical combs may result in BER fluctuations of the sliding test band in the wide WDM band even though they are utilized as the dummy WDM band. However, all the BER values are below the 20% soft-decision FEC threshold of 2.4 × 10^−2^, achieving successful data transmission with an aggregate capacity of 1.223 Pbps (net 1.02 Pbps) and a SE of 156.8 bit s^−1^ Hz^−1^ (net 130.7 bit s^−1^ Hz^−1^) over the 34 km 7-core RCF.

## Discussion

An OAM fibre-optic transmission system with a total capacity of over 1 Pbps has been experimentally demonstrated for the first time. Implemented over a 34 km 7-core RCF with a cladding diametre of 180 µm supporting 80 available OAM mode channels each carrying 312 WDM channels, the OAM-SDM-WDM system achieves a raw (net) capacity of 1.223 (1.02) Pbps and a SE of 156.8 (130.7) bit s^−1^ Hz^−1^.

Noteworthily, the simultaneous weak coupling among fibre cores and amongst the non-degenerate OAM MGs within each core enables the use of small-scale 4 × 4 modular MIMO processing with very short tap lengths of 25, to compensate only the coupling between the nearly degenerate intra-MG modes. To put the results into perspective, a comparison of the computational complexity (RNCM/symbol for time-domain MIMO equalization calculated according to the equations and parametres listed in Supplement S1) can be made. The intra-MG 4 × 4 time-domain MIMO equalization needs only 100 RNCM/symbol, while if the 80 mode channels were fully coupled with the DGD given in Fig. 4 (around 410 ns if the inter-MG coupling remains weak after the 34 km transmission), a full 80 × 80 MIMO would be required with a complexity of 8 × 10^5^ RNCM/symbol, more than three orders of magnitude higher than our scheme and practically impossible to implement as it would be equivalent to more than 120 Peta floating point operations per second (FLOPS) (about a quarter of the computational power of Fugaku, the world’s current top supercomputer!) per channel, assuming 6 FLOPS are needed per complex-valued multiplication.

Although the small MIMO size allows time-domain equalization (TDE) to be used in our experiment, comparisons can also be made in the case of frequency-domain equalization (FDE) by calculating the RNCM/symbol according to the equations presented in Supplement S1. If the 80 mode channels were fully coupled with the DGD as given in Fig. 4, the FDE MIMO complexity would be ~ 137 RNCM/symbol, whereas in our scheme, the value would be ~ 16, a saving of nearly 10 times which is still very significant. Furthermore, the buffer memory size that should cover the number of the samples required for the Fast Fourier Transform (*N*_FFT_) in FDE in our case can be only 64 per mode channel (here assuming the *N*_FFT_ equals twice the tap length of 25 rounded up to the nearest power of 2) or ~ 5120 in total to cover the very small intra-MG DGD, compared to ~ 2.6 × 10^6^ in the case of the full 80 × 80 MIMO that would have to cover the full DGD, showing a difference of more than two orders of magnitude.

With our scheme, the up-scaling of the receiver can therefore be implemented in a modular fashion which will only require more of the same 4 × 4 MIMO DSP modules, as more OAM mode groups are added in the fibre either by supporting more OAM MGs in each core or by increasing the number of cores, under the precondition of maintaining low coupling between the MGs—previously we have already demonstrated low mode coupling and attenuation levels in single core RCFs that sustained 100 km transmission^19^. Therefore, the scheme demonstrates significant potentials for up-scaling of transmission capacity per optical fibre while keeping ultra-low MIMO complexity, and consequently, low cost and low power consumption, by exploiting the uniquely excellent characteristics of OAM modes in ring core optical fibres over distances of tens of kilometres (e.g., the metro, or inter-data centre links, etc.) where weak coupling among the non-degenerate modes within each fibre core can be maintained and the challenging in-line optical amplification towards FM-MCFs is not required.

Although the cost of future SDM systems depends on many factors including the fibres, the (de)multiplexers and the optical amplifiers, these are likely to be similar for RCF-OAM and FMF-LP schemes, with relatively simpler DEMUX and more equalized gain at the amplifiers slightly favouring the RCF-OAM scheme^17^. Assuming these factors being largely equal, the low and constant MIMO complexity in the RCF-OAM scheme could be an important differentiator when compared to other SDM schemes.

Currently, the mode multiplexing in our 7-core OAM MUX module is simply power combining, whose insertion loss scales up with the number of MG used. With a small number of modes to be multiplexed, such a scheme is acceptable. When many more mode channels are involved, the loss from power combining will become dominant. Advanced multi-core spiral transformation^39^ or multi-plane light conversion^40^ should be more power-efficient. MUX/DEMUX modules in a more integrated form would be highly desirable, but these will have to convert densely packed input Gaussian beams from/to a complex 2D input fibre array, which will be a significant optical challenge.

## Materials and methods

### Electrical signals modulated on the test and dummy WDM channels

The odd and even groups of the test band (shown in Fig. 3) are independently modulated by two 24.5-GBaud QPSK signals from a four-channel AWG through in-phase/quadrature (I/Q) modulators. The sampling rate of the AWG is set to 64 GSa s^−1^ and the data sequence is the pseudo-random binary sequence with a pattern length of 2^18^-1. The electrical signals are digitally pre-shaped by a Nyquist filter (raised cosine filter) with a roll-off factor of 0.01 to adapt to the 25 GHz WDM grid.

As shown in Fig. 3, the dummy band is data-loaded via one IQ modulator driven by two electrical signals that are de-correlated replicas of the ones used to modulate the even group of the test band, generating 24.5-Gbaud QPSK signals.

### 7-core OAM MUX modules

**Fig. 7:**
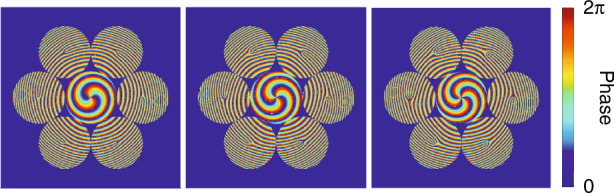
The vortex phase plate diagrams for generating 7-core OAM modes. Each represents topological charge of *l* = +2, +3, and +4.

The 7-core OAM MUX module in our experiment is realized using free-space optics in which low inter-core and inter-MG crosstalk can be achieved simultaneously. As shown in Figs. 3, 7 optical beams from a hexagonally packed 7-core SMF spliced with a fan-in device are imaged onto a spatial light modulator (SLM) after passing through an optical lens and a linear polarizer and converted into 7 OAM beams with topological charge +*l* (*l* = 2, 3, or 4) by the SLM. A phase-only modulation mask is programmed on the SLM, which consists of hexagonally configured VPP diagrams around their local optical axis, superimposed with a global radial quadratic phase distribution, as shown in Fig. 7, to precisely generate one specific OAM mode in each core of the 7-core RCF. The principle of the coupling scheme can be found in the paper^41^ and the detailed parametres are listed in Supplement S12.

By the same method, 7 OAM beams with topological charge *−l* can be produced in another branch. Then the two branches of the generated OAM beams are adjusted into orthogonal polarization states (*<+l, X*> and <*−l, Y*>*, X,* and *Y* representing horizontal and vertical polarization respectively) by inserting a half-wave plate (HWP) in one branch. Therefore, they can be combined using a polarization beam combiner (PBC) rather than a beam splitter, since the latter will introduce an additional 3 dB insertion loss that impacts on the power budget of the transmission system. After circular polarization conversion through a QWP, the OAM beams are injected into the polarization multiplexing module, which consists of a polarization beam splitter (PBS), an optical de-correlation path with two lenses and a PBC (see the ‘polarization mux.’ module coloured in green of Fig. 3), to produce the four intra-MG OAM modes <*+l, R*>, <*+l, L*>, <*−l, R*> and <*−l, L*>, where *R* and *L* refer to the left- and right-handed circular polarization, respectively. Here the two lenses in the optical de-correlation path have the same focal length and are in a 4-f configuration, so that the beam pitch and the optical axes of the seven-core OAM beams after the de-correlation-path transmission can be unchanged, which will be beneficial to efficient combining of the two bunches of OAM beams with and without optical de-correlations and thus high coupling efficiency to the 7-core RCF. The focal distance of the lenses utilized in the experiment is selected to be 100 mm, and 4-f system introduces a path length difference of 400 mm between two polarization branches, leading to a time delay of 1.3 ns or a symbol delay of 32 at a symbol rate of 24.5GBaud. Multiplexing of three weakly-coupled OAM MGs with topological charge *|l*| = 2, 3, and 4 can be realized by power combining multiple such 7-core OAM MG generation modules before the polarization multiplexing.

## Supplementary information

1-Pbps Orbital Angular Momentum Fibre-optic Transmission
