# Supplementary material for: 1-Pbps orbital angular momentum fibre-optic transmission
Source: Light Sci Appl. 2022 Jul 5;11:202. doi: 10.1038/s41377-022-00889-3 (PMC9256723; doi:10.1038/s41377-022-00889-3)
Supplement: Supplementary file 1 — 1-Pbps Orbital Angular Momentum Fibre-optic Transmission [file 41377_2022_889_MOESM1_ESM.docx]

**Supplementary Information for “1-Pbps Orbital Angular Momentum Fibre-optic Transmission”**

*Junyi Liu^1,†^, Jingxing Zhang^1,†^, Jie Liu^1,*^, Zhenrui Lin^1^, Zhenhua Li^1^, Zhongzheng Lin^1^, Junwei Zhang^1^, Cong Huang^1^, Shuqi Mo^1^, Lei Shen^2^, Shuqing Lin^1^, Yujie Chen^1^, Ran Gao^3^, Lei Zhang^2^, Xiaobo Lan^2^, Xinlun Cai^1^, Zhaohui Li^1^, and Siyuan Yu^1,*^*

*^1^State Key Laboratory of Optoelectronic Materials and Technologies, School of Electronics and Information Technology, Sun Yat-Sen University, Guangzhou 510006, China.*

*^2^Yangtze Optical Fibre and Cable Joint Stock Limited Company, State key Laboratory of Optical Fibre and Cable Manufacture technology No.9 Guanggu Avenue, Wuhan, Hubei, China.*

*^3^School of Information and Electronics, Beijing Institute of Technology, Beijing 100081, China.*

*† These authors contributed equally.*

**Corresponding author:* [*liujie47@mail.sysu.edu.cn*](mailto:liujie47@mail.sysu.edu.cn)*;* [*yusy@mail.sysu.edu.cn*](mailto:yusy@mail.sysu.edu.cn)

Contents

[**S1. MIMO-complexity calculations of space-division multiplexed systems** 1](#_Toc105576436)

[**S2. Comparisons of different kinds of SDM transmission systems** 3](#_Toc105576437)

[**S3. The effective refractive indices of the 7-core RCF across C and L Band** 4](#_Toc105576438)

[**S4. Measurement of mode-dependent attenuation in the 34-km 7-core RCF based on OTDR** 5](#_Toc105576439)

[**S5. A Cut-Off method for measuring wavelength-dependent attenuation of the 34-km 7-core RCF** 7](#_Toc105576440)

[**S6. Measurement setup of differential-group delay using a vector network analyzer** 8](#_Toc105576441)

[**S7. Evaluation of BER degradation resulted from fibre nonlinearity in the OAM-SDM transmission experiment** 8](#_Toc105576442)

[**S8. BER vs. received optical power of the OAM-SDM-WDM experiment system** 9](#_Toc105576443)

[**S9. Measurement of inter-MG crosstalk and the inter-core crosstalk of the entire transmission system and the 34-km 7-core RCF** 10](#_Toc105576444)

[**S10. BER vs. OSNR in the single-wavelength transmission scenario** 15](#_Toc105576445)

[**S11. Tap-weight absolute values recovered after transmission in some typical spatial/wavelength channels** 16](#_Toc105576446)

[**S12. Principle of the ‘7-to-7’ coupling scheme** 17](#_Toc105576447)

[**References** 18](#_Toc105576448)

# **S1. MIMO-complexity calculations of space-division multiplexed systems**

1. *The complexity of time-domain MIMO equalization for single/multi-carrier modulations*

In the space division multiplexed (SDM) transmission systems, the filter taps in a digital MIMO equalizer needed to cover the differential mode delay (DMD) can be expressed as^1, 2^:

$$N_{\mathrm{MD}}=\Delta T_{\mathrm{MD}}r_{\mathrm{os}}R_{\mathrm{s}}$$

where $\Delta T_{\mathrm{MD}}$ is DMD covering all coupled modes, $r_{\mathrm{os}}$is the oversampling factor (usually equals to 1 or 2), and $R_{\mathrm{s}}$is the data symbol rate.

In the SDM transmission systems with single-carrier modulation, the complexity of time-domain *D* × *D* MIMO equalizers can be expressed as the required number of complex multiplications (RNCM) per symbol^1^:

$$RNCM_{\mathrm{per} \mathrm{symbol}, \mathrm{sc}, \mathrm{TDE}}=r_{\mathrm{os}}N_{\mathrm{MD}}D$$

where *D* is MIMO dimension (usually equals to the number of coupled spatial channels). Considering different transmission capacities may be supported in various SDM systems, RNCM per unit capacity should be calculated to give an equitable comparison:

$$RNCM_{\mathrm{per} \mathrm{capacity}, \mathrm{sc},\mathrm{TDE}}=\frac{RNCM_{\mathrm{per} \mathrm{symbol},\mathrm{sc}, \mathrm{TDE}}R_{\mathrm{s}}MW}{N_{\mathrm{bit}}R_{\mathrm{s}}MW}=\frac{r_{\mathrm{os}}N_{\mathrm{MD}}D}{N_{\mathrm{bit}}}=RNCM_{\mathrm{per} \mathrm{bit}, \mathrm{sc},\mathrm{TDE}}$$

where$N_{\mathrm{bit}}$is the number of bits per symbol related to the modulation formats. $M$ and $W$refer to the number of mode and wavelength channels in the SDM transmissions, respectively. Thus$R_{\mathrm{s}}MW$denotes the totally transmitted symbol number per second, while $N_{\mathrm{bit}}R_{\mathrm{s}}MW$is the system transmission capacity.

In the SDM transmission systems with multi-carrier modulation, RNCM per unit capacity of the time-domain MIMO equalization can be expressed as^3^:

where $N_{\mathrm{FFT}}$is the size of FFT/IFFT, and$N_{\mathrm{sc}}$is the number of sub-carriers.

1. *The complexity of frequency-domain MIMO equalization for single/multi-carrier modulations*

As an alternative, frequency-domain MIMO equalizations using the Fast Fourier Transform (FFT) algorithm have also been utilized in the SDM transmission systems to cope with crosstalk among spatial channels, showing low calculation complexity ^1, 4-5^, In the *D* × *D* MIMO frequency-domain equalizers (FDEs) with single-carrier modulation, RNCM per unit capacity here can be expressed as^1, 2^:

$$RNCM_{\mathrm{per} \mathrm{capacity}, \mathrm{sc}, \mathrm{FDE}}=\text{ }\frac{r_{\mathrm{os}}[N_{\mathrm{FFT}}\log_{2}(N_{\mathrm{FFT}})+N_{\mathrm{FFT}}D]}{(N_{\mathrm{FFT}}-N_{\mathrm{MD}}+1)N_{\mathrm{bit}}}=RNCM_{\mathrm{per} \mathrm{bit}, \mathrm{sc}, \mathrm{FDE}}$$

where $N_{\mathrm{FFT}}$ is the number of samples of each FFT block. It is often a value with the form of 2*^n^* (*n* is a positive integer) more than $N_{\mathrm{MD}}$.

As for multi-carrier modulation, RNCM per unit capacity in the *D* × *D* MIMO FDEs with multi-carrier modulation can be written as^3^:

$$RNCM_{\mathrm{per} \mathrm{capacity}, \mathrm{mc}, \mathrm{FDE}}=\frac{N_{\mathrm{FFT}}\log_{2}(N_{\mathrm{FFT}})+N_{\mathrm{sc}}D}{N_{\mathrm{sc}}N_{\mathrm{bit}}}=RNCM_{\mathrm{per} \mathrm{bit}, \mathrm{mc}, \mathrm{FDE}}$$

1. *MIMO Complexity calculations shown in Fig. 1 of the main text*

In the Pbps-level capacity SDM transmission systems with single-carrier modulation, time-domain *D* × *D* MIMO equalizers were utilized according to the description in Ref. [2-5,7-11] in the main text. Based on their provided parametres such as the number of time-domain filter taps, the number of coupled spatial channels, modulation formats, etc., the RNCMs per unit capacity are calculated according to Eq. (2) and Eq. (3). Here noted that the oversampling rate in the calculation is set to 1 unless the oversampling in MIMO equalization is specially noted in the paper. As for the Pbps-level capacity SDM transmission systems with multi-carrier modulation, RNCM per unit capacity of the frequency-domain MIMO equalization is calculated according to Eq. (6) based on the parametres presented in Ref. [6] of the main text.

The fibre transmission lengths are not considered in the MIMO complexity comparisons shown in Fig. 1 of the main text. Actually, the distance in our OAM-SDM-WDM transmission experiment is the longest among the SDM transmission systems with a capacity over 1-Pbps based on few-mode multi-core fibres (FM-MCFs) or multi-mode single-core fibres (MM-SCFs). As for the schemes based on single-mode multi-core fibres (SM-MCFs), only a few filter taps (normally 10 to 20 taps required in most demonstrations) can cover polarization mode dispersion (PMD) in the SMF core with even thousands of kilometres. Therefore, the MIMO complexity in the Pbps-level capacity SDM schemes based on SM-MCFs with transmission distance no more than hundreds of kilometres can be considered nearly independent of the transmission distance.

1. *Parametres of calculations in the section ‘Conclusions and discussions’ of the main text*

**Table S1** Parametres used in the calculations for 34-km OAM-SDM systems with modular 4 × 4 or full 80 × 80 MIMO equalization

|  | Time-domain | |  | Frequency-domain | |
| --- | --- | --- | --- | --- | --- |
|  | 4 × 4 MIMO | 80 × 80 MIMO |  | 4 × 4 MIMO | 80 × 80 MIMO |
| $\Delta T_{\mathrm{MD}}$ | / | 410 ns |  | / | 410 ns |
| $R_{\mathrm{s}}$ | 24.5 GBaud | 24.5 GBaud |  | 24.5 GBaud | 24.5 GBaud |
| *D* | 4 | 80 |  | 4 | 80 |
| $N_{\mathrm{bit}}$ | 2 | 2 |  | 2 | 2 |
| $r_{\mathrm{os}}$ | 1 | 1 |  | 1 | 1 |
| $N_{\mathrm{MD}}$ | 25 | 10045 |  | 25 | 10045 |
| $N_{\mathrm{FFT}}$ | / | / |  | 64 | 32768 |
| RNCM/symbol | 100 | 803600 |  | 16 | 137 |
| FLOPS/mode channel | 14.7 Tera | 118 Peta |  | 2.4 Tera | 20.1 Tera |
| Buffer size | / | / |  | 5120 | 2621440 |

# **S2. Comparisons of different kinds of SDM transmission systems**

In this section, detailed parametres of the reported SDM transmission systems based on multi-core few-mode fibres with capacity more than 1 Pbps and the OAM-based MDM system in recent works are summarized, as shown in Table S2 and S3, respectively.

**Table S2** Comparison of the reported transmission systems based on multi-core few-mode fibres with capacity more than 1 Pbps

| Ref. (year) | Cores × Modes | Cladding diametre (μm) | Distance (km) | MIMO complexity | Modulation format | BER (Q-factor) | Net SE (bit s^-1^ Hz^-1^) | Net capacity (Tbit s^-1^) |
| --- | --- | --- | --- | --- | --- | --- | --- | --- |
| [4] (2019) | 4 × 3 | 160 | 3.37 | 6 × 6, 751 taps | DP-256 QAM | Below 1.41 × 10^-3^ (2-5 dB) | 130.65 | 1202 |
| [6] (2012) | 12 × 1 + 2 × 3 | 216 | 3 | 6 × 6 | DP-32 QAM | Below 3.8 × 10^-3^ | 109 | 1050 |
| [8] (2020) | 38 × 3 | 312 | 13 | 6 × 6, 249 taps | DP-64/256 QAM | Below 2.2 × 10^-5^ | 1158.7 | 10660 |
| [9] (2015) | 19 × 6 | 318 | 9.8 | 12 × 12, 1000 taps | DP - QPSK | Below 2.3 × 10^-2^ | 456 | 2050 |
| [10] (2018) | 19 × 6 | 267 | 11.31 | 12 × 12, 200-300 taps | DP-16/64 QAM | Below 3.4 × 10^-6^ (Q > 4.98) | 1099.9 | 10160 |
| This work (2022) | 7 × 6 | 180 | 34 | 4 × 4, 25 taps | DP - QPSK | Below 2.4 × 10^-2^ | 130.76 | 1020 |

DP: dual-polarization.

The associated References label are from the Ref. [4,6,8,9,10] of the main text.

**Table S3** Data transmission of the OAM-based MDM system in recent works

| Fibre Types | Loss (dB km^-1^) | Signal and MIMO DSP | Number of MDM/WDM Channels | Capacity (Tbit s^-1^) | Distance (km) | Number of core | Year | Ref. |
| --- | --- | --- | --- | --- | --- | --- | --- | --- |
| Vortex fibre | 1.6 | 16QAM, MIMO free | 2/10 | 1.6 | 1.1 | 1 | 2013 | [15] |
| Graded index | – | QPSK, 2 × 2 MIMO | 4/1 | 0.256 | 1.4 | 1 | 2016 | [33] |
| Graded index | – | QPSK, MIMO free | 2/16 | 0.64 | 18 | 1 | 2018 | [21] |
| Air core fibre | – | QPSK, MIMO free | 12/60 | 10.56 | 1.2 | 1 | 2018 | [29] |
| Graded index | 1 | QPSK, 4 × 4 MIMO | 8/10 | 5.12 | 10 | 1 | 2018 | [20] |
| Graded index | 1 | 8QAM, MIMO free | 2/112 | 8.44 | 18 | 1 | 2018 | [22] |
| RIP-modulated | 0.31 | QPSK, 4 × 4 MIMO | 8/10 | 2.56 | 50 | 1 | 2019 | [18] |
| RIP-modulated | 0.21 | QPSK, 4 × 4 MIMO | 8/10 | 2.56 | 100 | 1 | 2020 | [19] |
| RIP-modulated | 0.29 | QPSK, 4 × 4 MIMO | 12/30 | 11.52 | 25 | 1 | 2021 | [16] |
| MC-RCF | 0.29 | QPSK, 4 × 4 MIMO | 12/312 | 1020 | 34 | 7 | 2022 | This  work |

RIP: refractive index profile.

The associated References label are from the Ref. [15,16,18-22,29,33] of the main text.

# **S3. The effective refractive indices of the 7-core RCF across C and L Band**

The modal effective refractive indices of all guided modes within C + L band of optical wavelengths (from 1530 nm to 1610 nm) are calculated using a commercial finite element module (COMSOL Multiphysics) based on the designed RIP. As plotted in Fig. S1. the differential effective refractive index (Δ*n*_eff_) between adjacent OAM mode groups (MGs) with topological charge |*l*| ≥ 1 is greater than 1.5 × 10^−3^ across C + L band, while the 4 modes in each MG (+/- *l*, each carrying two orthogonal polarizations) are highly degenerate, as shown in Fig. S2, where four wavelengths (1540 nm, 1560 nm, 1580 nm, and 1600 nm) are here presented for reference.

Fig. S1 Modal effective refractive index versus wavelength in the fibre core.

Fig. S2 Effective refractive indices of eigen-modes at wavelengths of **a** 1540 nm, **b** 1560 nm, **c** 1580 nm, and **d** 1600 nm.

# **S4. Measurement of mode-dependent attenuation in the 34-km 7-core RCF based on OTDR**

The attenuation of each guided OAM MG for each core of the 7-core RCF at 1550 nm is measured utilizing an optical time-domain reflectometre (OTDR) as shown in Fig. S3. The light from OTDR is launched into one of the cores of the 7-core RCF through a vortex phase-plate (VPP) with topological charge *l* = 0 to +4 to selectively excite the corresponding OAM modes of MG |*l*| = 0 to 4. The attenuation measurement is repeated for each fibre core and the measured results for core #0 to core #6 are depicted in Figs. S4a-g, respectively.

Fig. S3 OTDR based mode attenuation measurement setup of the 7-core RCF at 1550 nm. OTDR: optical time-domain reflectometre; Col.: collimator; VPP: vortex phase plate; QWP: quarter-wave plate.

Fig. S4 Measured mode-dependent attenuation for **a** core #0, **b** core #1, **c** core #2, **d** core #3, **e** core #4, **f** core #5, and **g** core #6 of the 7-core RCF.

# **S5. A Cut-Off method for measuring wavelength-dependent attenuation of the 34-km 7-core RCF**

The fibre attenuation is measured utilizing a cut-off method to evaluate the fibre attenuation variation across the WDM transmission band of fibre core #0 and core #4, as the setup shown in Fig. S5. The beam from the wavelength-tunable laser is converted to OAM modes of MG *l* = +3 by a spatial light modulator (SLM) after passing through a lens and linear polarizer (LP). Then it is coupled, through a collimator, into one selected core of the 34-km 7-core RCF. The wavelength of the light source is stepped (at a step-size of 2 nm) from 1540 nm to 1600 nm while recording the input and corresponding output power$P_{0}^{i}(\lambda)$and$P_{0}^{o}(\lambda)$using a power metre. The fibre is then cut off (as quickly and stably as possible), leaving a tail with a length of around 1 metre. The measurement is repeated to record the input power$P_{1}^{i}(\lambda)$and output power$P_{1}^{o}(\lambda)$. The attenuation coefficients measured using this method can be expressed as:

$$\alpha(\lambda)=-\frac{[P_{0}^{o}(\lambda)-P_{0}^{i}(\lambda)]-[P_{1}^{o}(\lambda)-P_{1}^{i}(\lambda)]}{L_{0}-L_{1}}$$

where *L*_0_ = 34 km, *L*_1_0.001 km.

Fig. S5 Experiment setup for measuring attenuation coefficient using the cut-off method. PC: polarization controller; FI: fan in; LP: linear polarizer; SLM: spatial light modulator; QWP: quarter-wave plate; Col.: collimator.

As shown in Fig. S6, core #0 and core #4 exhibit an average fibre attenuation of 0.28 dB km^-1^ and 0.30 dB km^-1^, respectively. The wavelength-dependent attenuation of the two cores is within 0.02 dB km^-1^ from 1540 nm to 1600 nm.

Fig. S6 Measured mode attenuation for core #0 & core #4.

# **S6. Measurement setup of differential-group delay using a vector network analyzer**

The differential-group delay (DGD) of each guided OAM MG for each core of the 7-core RCF is measured by an impulse response measurement setup based on a vector network analyzer (VNA)^6, 7^, as shown in Fig. S7. The radio-frequency (RF) signals with a frequency range from 10 MHz to 2 GHz from the VNA modulate the optical carrier via a Mach-Zehnder modulator (MZM). The optical carrier is generated by a tunable laser at one of the wavelengths of 1540 nm, 1560 nm, 1580 nm, and 1600 nm. After amplification by an EDFA, the light beam is incident on the SLM to generate the OAM beam *l* = +*n*. The generated OAM beam is converted to a circular polarization state by a QWP before being collimated into one of the cores of the 7-core RCF. The light after single-core transmission passes through a VPP with *l* = -*n*, therefore converting the corresponding OAM beam *l* = +*n* into a Gaussian beam. The resulting Gaussian beam is collimated into an SMF pigtail for optical amplification and detection by a photodetector (PD). Finally, the detected RF signal is fed back to the VNA for impulse response measurement. After obtaining the impulse response, the DGD of each guided OMA MG can be calculated by its time delay relative to OAM MG |*l*| = 0. The DGD measurement is repeated for each fibre core and each wavelength.

Fig. S7 VNA based impulse response measurement setup for determining DGD in a fibre. VNA: vector network analyzer; PC: polarization controller; MZM: Mach-Zehnder modulator; EDFA: erbium doped fibre amplifier; FI: Fan in; LP: linear polarizer; SLM: spatial light modulator; QWP: quarter-wave plate; Col.: collimator; VPP: vortex phase plate; PD: photodetector.

# **S7. Evaluation of BER degradation resulted from fibre nonlinearity in the OAM-SDM transmission experiment**

As 24960 channels (80 mode channels × 312 wavelength channels) are [simultaneously](http://www.youdao.com/w/simultaneously/#keyfrom=E2Ctranslation) transmitted in the 34 km 7-core RCF with a total optical power of 19.8 dBm launched to the fibre, it should be concerned whether the nonlinear effects significantly deteriorate the system BER performance in this case. In both of the single- and multi-wavelength scenarios of the OAM-SDM transmission system over 34-km 7-core RCF (setup similar with that shown in Fig. 3 of the main text), the bit-error rates (BERs) OAM MG |*l*| = 3 in the central fibre core at different optical power launched to the fibre are experimentally measured with the existence of both inter-core and inter-mode-group (MG) crosstalk, as the results shown in Fig S8. It can be seen that the BERs deteriorate as the optical power launched to the fibre increases, when the launched optical power is more than 16 dBm, due to the fibre nonlinearity. However, the BERs are still below the 20% soft-decision FEC threshold of 2.4 × 10^−2^ for both of the single- and multi-wavelength transmission scenarios, even though the totally launched optical power achieves 24 dBm, showing the fibre nonlinearity can be acceptable in this case. Here we note that only one OAM MG with high power is tested for simplicity. We believe fibre nonlinearity can be alleviated when such high power is allocated in different OAM MGs due to the large inter-MG differential delay in each fibre core.

Fig. S8 BER versus total optical power of OAM MG |*l*| = 3 launched to the Core #0.

# **S8. BER vs. received optical power of the OAM-SDM-WDM experiment system**

Fig. S9 The measured BER and corresponding received optical power of the OAM-MDM-SDM transmission.

To evaluate the power budget at the receiver of the OAM-SDM-WDM experimental system, the BER versus the received optical power at the input port of the pre-amplifier located before the coherent optical receiver is measured. As shown in Fig. S9, the measured BER values of all the OAM modes in the multi-wavelength transmission scenario of fibre core #0 and core #6 can be below the 20% soft-decision FEC threshold of 2.4 × 10^−2^ with all the inter-core and inter-MG crosstalk. It is concluded that the received optical power at the input port of the pre-amplifier should be above -37 dBm.

# **S9. Measurement of inter-MG crosstalk and the inter-core crosstalk of the entire transmission system and** **the 34-km 7-core RCF**

The inter-MG crosstalk (XT) within the same fibre core as well as the inter-core XT of the transmission system including the 34-km 7-core RCF, the OAM MUX and DEMUX are experimentally characterized based on power measurements. Meanwhile, the inter-MG coupling coefficient of the 34-km 7-core RCF are also characterized using integral calculation based swept-wavelength interferometry measurement.

1. *The measurement of inter-MG crosstalk of the entire SDM transmission system*

Three OAM MGs with the topological charge of 2, 3 and 4, after propagation in one 34 km long fibre core, are split into two branches, with each branch passing through a commercial VPP with opposite topological charge *l* and -*l* respectively (see Fig. 3 in the main text). Only OAM MG whose topological charge equals |*l*| can be demultiplexed and converted to Gaussian beams and coupled into two SMFs. The value of inter-MG crosstalk in the system is obtained in the following way. Firstly, the average power$P_{\left|l\right|}$(unit: dBm) of the demultiplexed OAM MG |*l*| at the end of the two SMFs is recorded. Then the transmitter is switched to another MG |*m*| (*m* ≠ *l*) and its average power $P_{\left|m\right|}$is recorded at the output of the same SMFs. The crosstalk from OAM MG |*m*| to OAM MG |*l*| is calculated as:

$$XT_{\left|l\right|,\left|m\right|}=P_{\left|m\right|}-P_{\left|l\right|}$$

where *m*, *l* = 2, 3 and 4. The detailed results of the measured inter-MG crosstalk of all side cores at the wavelengths of 1555 nm, 1565 nm, 1575 nm and 1585 nm are illustrated in Fig. S10, which show that all the measured inter-MG XTs are below -12 dB.

Fig. S10 The measured inter-MG crosstalk among three high-order OAM MGs of all cores at **a** 1555 nm, **b** 1565 nm, **c** 1575 nm, and **d** 1585 nm over 34-km 7-core RCF transmission system.

1. *The measurement of inter-core crosstalk of the transmission system*

To measure the inter-core crosstalk, each time one OAM MG (|*l*| = 2, 3 and 4) is coupled into a selected core #*n* (*n* = 0-6), whose power at the output is $P_{\mathrm{Im}}^{l}$, while the output power detected from other cores are $P_{n^{*}}^{|l|}$(*n^*^*≠ *n*). The inter-core crosstalk of MG |*l*| (2, 3 and 4) between core #*n* and core #*n*^*^ is then recorded as:

$$XT_{nn^{*}}^{|l|}=P_{n^{*}}^{|l|}-P_{n}^{|l|}$$

The detailed results of the measured inter-core crosstalk of all side cores at the wavelengths of 1555 nm, 1565 nm, 1575 nm and 1585 nm are illustrated in Fig. S11, which show that all the measured inter-MG XTs are below -20 dB.

Fig. S11 The measured inter-core crosstalk among the other cores of all cores at **a** 1555 nm, **b** 1565 nm, **c** 1575 nm, and **d** 1585 nm over 34-km 7-core RCF transmission system.

*C．Characterization of inter-MG coupling coefficient of the 34-km 7-core RCF*

The measurement step is illustrated in Fig S12. The light beam is generated by a tunable laser whose wavelength scan span and the sweeping speed are set to 0.2 nm and 2000 nm s^-1^, respectively, and then be divided into two paths by a 50:50 optical coupler (OC). In the upper path utilized as the probe arm, the optical signal is amplified by an EDFA, converted to OAM mode with topological charge *l* (where *l* = -4, -3, -2, +2, +3 or +4) by an SLM, and finally collimated into the 34-km 7-core RCF. After fibre transmission, the received OAM beams are collimated, converted back to linearly polarization using a quarter-wave plate (QWP) and split into two branches, each of which is converted into Gaussian beam by the VPP with topological charge +*l* or -*l* (where *l* = 2, 3 or 4) and subsequently coupled into an SMF-pigtailed dual-polarization integrated coherent receiver (ICR). Finally, the detected electrical signals are digitized and stored by a 4-channel real-time oscilloscope (OSC) for off-line processing to obtain the impulse responses. The impulse response matrix for all supported OAM modes of core #0 and core #6 of the 34-km 7-core RCF at the wavelength of 1545 nm, 1565 nm, 1585 nm and 1605 nm can be obtained (the measured impulse responses of the modes supported in core #6 at 1585 nm are depicted in Fig. S14 for reference). Temporal integrals of the excited mode peak and the DGD plateau (as shown in Fig. S13) are respectively calculated, and the ratio of integrals of the DGD plateau and the excited mode peak represents the accumulated inter-MG XT of 34-km 7-core RCF whose values are shown in Table S4. (Specific calculation methods can be obtained from [7]).

Fig. S12 SWI-based impulse response measurement setup for determining mode coupling in a fibre. EDFA: erbium doped fibre amplifier; PC: polarization controller; Col.: collimator; LP: linear polarizer; SLM: spatial light modulator; QWP: quarter-wave plate; BS: beam splitter; VPP: vortex phase plate; ICR: integrated coherent receiver; OSC: oscilloscope.

Fig. S13 The measured impulse responses in the weak mode coupling case for **a** mode 1 excitation & mode 1 detection and **b** mode 1 excitation & mode 2 detection. The red areas represent the total power of strong launched mode/ mode coupling.

Fig. S14 The SWI-measured impulse response matrix of core #6 at 1585 nm.

**Table. S4** Experimental characterization of the crosstalk between adjacent MG using SWI-based impulse response measurement setup

| Relative power (dB km^-1^) @ 1545 nm | | | Received MG | | |
| --- | --- | --- | --- | --- | --- |
|  |  |  | \|*l*\| = 2 | \|*l*\| = 3 | \|*l*\| = 4 |
| Launched  MG | Core 0 | |*l*| = 2 | 0 | -31.65 | -36.25 |
|  |  | \|*l*\| = 3 | -30.78 | 0 | -30.81 |
|  |  | \|*l*\| = 4 | -35.96 | -30.20 | 0 |
|  | Core 6 | \|*l*\| = 2 | 0 | -31.39 | -34.67 |
|  |  | \|*l*\| = 3 | -29.95 | 0 | -30.54 |
|  |  | \|*l*\| = 4 | -34.78 | -30.60 | 0 |
| Relative power (dB km^-1^) @ 1565 nm | | | Received MG | | |
|  |  |  | \|*l*\| = 2 | \|*l*\| = 3 | \|*l*\| = 4 |
| Launched  MG | Core 0 | |*l*| = 2 | 0 | -30.43 | -35.83 |
|  |  | \|*l*\| = 3 | -30.43 | 0 | -29.16 |
|  |  | \|*l*\| = 4 | -35.13 | -30.92 | 0 |
|  | Core 6 | \|*l*\| = 2 | 0 | -29.39 | -34.00 |
|  |  | \|*l*\| = 3 | -31.69 | 0 | -31.68 |
|  |  | \|*l*\| = 4 | -33.01 | -31.60 | 0 |
| Relative power (dB km^-1^) @ 1585 nm | | | Received MG | | |
|  |  |  | \|*l*\| = 2 | \|*l*\| = 3 | \|*l*\| = 4 |
| Launched  MG | Core 0 | |*l*| = 2 | 0 | -31.76 | -32.32 |
|  |  | \|*l*\| = 3 | -29.40 | 0 | -31.67 |
|  |  | \|*l*\| = 4 | -33.98 | -29.16 | 0 |
|  | Core 6 | \|*l*\| = 2 | 0 | -31.99 | -33.72 |
|  |  | \|*l*\| = 3 | -30.39 | 0 | -29.48 |
|  |  | \|*l*\| = 4 | -30.96 | -29.50 | 0 |
| Relative power (dB km^-1^) @ 1605 nm | | | Received MG | | |
|  |  |  | \|*l*\| = 2 | \|*l*\| = 3 | \|*l*\| = 4 |
| Launched  MG | Core 0 | |*l*| = 2 | 0 | -29.95 | -32.43 |
|  |  | \|*l*\| = 3 | -29.30 | 0 | -28.48 |
|  |  | \|*l*\| = 4 | -32.23 | -29.55 | 0 |
|  | Core 6 | \|*l*\| = 2 | 0 | -30.86 | -34.80 |
|  |  | \|*l*\| = 3 | -31.83 | 0 | -30.10 |
|  |  | \|*l*\| = 4 | -34.51 | -30.49 | 0 |

# **S10. BER vs. OSNR in the single-wavelength transmission scenario**

Fig. S15 plots the curves of BER versus OSNR of some selectively measured fibre cores/wavelengths, to give a performance comparison with that of the central core (Core #0) at 1540 nm shown in Fig. 6 of the main text. In conclusion, for the 7% soft-decision FEC threshold of 3.8 × 10^-3^, the OSNR penalty values between the cases with and without crosstalk are around 8 - 9 dB in the three cases shown in Fig. S15. The measured BERs can be below the 20% soft-decision FEC threshold of 2.4 × 10^-2^ with inter-MG crosstalk and inter-core crosstalk when OSNR is over 16 dB.

Fig. S15 The measured BER vs. OSNR curves of core #0 at **a** 1600 nm, core #6 at **b** 1540 nm and **c** 1600 nm after 34-km 7-core RCF transmission.

# **S11. Tap-weight absolute values recovered after transmission in some typical spatial/wavelength channels**

To recover the signals from strongly coupled intra-MG mode channels after the 34-km 7-core RCF transmission, the modular 4 × 4 MIMO equalization is utilized, whose convergent absolute values of complex tap weights of the 16 FIR filters are updated by conventional constant modulus algorithm (CMA) after 80 iterations, as the results shown in Fig. S16 (Core #0) and Fig. S17 (Core #6). Each consists of results for mode channel recovery of OAM MG |*l*| = 2, 3 and 4 at the wavelength of 1540 nm and 1600 nm. It can be seen that the number of taps required in each time-domain FIR filter is only 25, which is sufficient to cover the differential mode delay (DMD) of the four OAM modes within one MG.

Fig. S16 The absolute values of tap weights in 16 FIR filters of 4 × 4 MIMO equalizers to equalize the four modes belonging to OAM MGs **a** |*l*| = 2, **b** |*l*| = 3, **c** |*l|* = 4 at 1540 nm, **d** |*l|* = 2, **e** |*l*| = 3, **f** |*l*| = 4 at 1600 nm of core #0.

Fig. S17 The absolute values of tap weights in 16 FIR filters of 4 × 4 MIMO equalizers to equalize the four modes belonging to OAM MGs **a** |*l*| = 2, **b** |*l*| = 3, **c** |*l|* = 4 at 1540 nm, **d** |*l|* = 2, **e** |*l*| = 3, **f** |*l*| = 4 at 1600 nm of core #6.

# **S12. Principle of the ‘7-to-7’ coupling scheme**

Fig. S18 Schematic diagram of the ‘7-to-7’ scheme for OAM mode excitation in the 7-core RCF.

Fig. S18 illustrates the schematic diagram of the accurate 7-core OAM mode excitation and coupling scheme used in our experiment. The signals are firstly launched into hexagonal-arranged 7-core SMF through the integrated fan-in module. Then the 7 hexagonal-arranged Gaussian beams emitted from the 7-core SMF are imaged onto the phase mask via a thin lens. The phase mask is made up of 7 regions (see Fig. 7 of the main text), each converting one Gaussian beam into an OAM beam as well as correcting the optical axis. Finally, the generated OAM beams are coupled into the 7-core RCF by another lens.

The parametres used in our experiment are given in Table. S5.

**Table. S5** Experimental parametres of the ‘7-to-7’ coupling scheme

| 7-core SMF | core pitch | μm | 42 |
| --- | --- | --- | --- |
|  | focal length $f_{1}$ | mm | 8 |
|  | scaling factor $-\frac{s_{1}^{'}}{s_{1}}$ |  | 62 |
| 7-core RCF | core pitch | μm | 50 |
|  | focal length $f_{2}$ | mm | 8 |
|  | scaling factor $-\frac{s_{2}^{'}}{s_{2}}$ |  | 52 |
| Phase mask | vortex order |  | +2, -2, +3, -3 +4, -4 |
|  | pitch of vortex phase | μm | 2600 |
|  | focal length of the Fresnel lens | mm | 226 |

# **References**

1. Arık, S., Askarov, D. & Kahn, J. M. Effect of mode coupling on signal processing complexity in mode-division multiplexing. *Journal of Lightwave Technology* **31**, 423-431 (2013).
2. Proakis, J. G. & Salehi, M. Digital communications, 5th ed. (McGraw-Hill, 2007).
3. Zhang, J. W. *et al*. Orbital-angular-momentum mode-group multiplexed transmission over a graded-index ring-core fibre based on receive diversity and maximal ratio combining. *Optics Express* **26**, 4243-4257 (2018).
4. Inan, B. *et al*. DSP complexity of mode-division multiplexed receivers. *Optics Express* **20**, 10859-10869 (2012).
5. Benvenuto, N. *et al*. Algorithms for communications systems and their applications. (Wiley, 2002).
6. Maruyama, R. *et al*. Relationship between mode coupling and fibre characteristics in few-mode fibres analyzed using impulse response measurements technique. *Journal of Lightwave Technology* **35**, 650–657 (2017).
7. Zhang, J. W. *et al*. Accurate mode-coupling characterization of low-crosstalk ring-core fibres using integral calculation based swept-wavelength interferometry measurement. *Journal of Lightwave Technology* **39**, 6479-6486 (2021).
